# Activation of Toll-like receptor 2 promotes mesenchymal stem/stromal cell-mediated immunoregulation and angiostasis through AKR1C1

**DOI:** 10.7150/thno.100327

**Published:** 2024-08-06

**Authors:** Jung Hwa Ko, Hyun Ju Lee, Chang Ho Yoon, Yoo Rim Choi, Jin Suk Ryu, Joo Youn Oh

**Affiliations:** 1 Laboratory of Ocular Regenerative Medicine and Immunology, Biomedical Research Institute, Seoul National University Hospital, 101 Daehak-ro, Jongno-gu, Seoul 03080, Korea.; 2 Department of Ophthalmology, Seoul National University College of Medicine, 103 Daehak-ro, Jongno-gu, Seoul 03080, Korea.

**Keywords:** Ado-keto reductase family 1 member C1, Cornea, Mesenchymal stem/stromal cell, Monocyte/macrophage, Toll-like receptor 2

## Abstract

**Background:** Mesenchymal stem/stromal cells (MSCs) maintain tissue homeostasis in response to microenvironmental perturbations. Toll-like receptors (TLRs) are key sensors for exogenous and endogenous signals produced during injury. In this study, we aimed to investigate whether TLRs affect the homeostatic functions of MSCs after injury.

**Methods:** We examined the expression of TLR2, TLR3 and TLR4 in MSCs, and analyzed the functional significance of TLR2 activation using single-cell RNA sequencing. Additionally, we investigated the effects and mechanisms of TLR2 and its downstream activation in MSCs on the MSCs themselves, on monocytes/macrophages, and in a mouse model of sterile injury-induced inflammatory corneal angiogenesis.

**Results:** MSCs expressed TLR2, which was upregulated by monocytes/macrophages. Activation of TLR2 in MSCs promoted their immunoregulatory and angiostatic functions in monocytes/macrophages and in mice with inflammatory corneal angiogenesis, whereas TLR2 inhibition attenuated these functions. Single-cell RNA sequencing revealed *AKR1C1*, a gene encoding aldo-keto reductase family 1 member C1, as the most significantly inducible gene in MSCs upon TLR2 stimulation, though its stimulation did not affect cell compositions. AKR1C1 protected MSCs against ferroptosis, increased secretion of anti-inflammatory cytokines, and enhanced their ability to drive monocytes/macrophages towards immunoregulatory phenotypes, leading to the amelioration of inflammatory corneal neovascularization in mice.

**Conclusion:** Our findings suggest that activation of TLR2-AKR1C1 signaling in MSCs serves as an important pathway for the survival and homeostatic activities of MSCs during injury.

## Introduction

Adult hematopoiesis in the bone marrow (BM) is a dynamic, demand-adapted process wherein hematopoietic stem and progenitor cells (HSPCs) undergo self-renewal and differentiation to generate mature blood cells of all different lineages in response to both external and internal cues 1. Particularly during injury, HSPCs engage emergency myelopoiesis (EM) pathways that drive the rapid production of myeloid cells essential for resolution of initiating insults at the expense of other lineages. Recent evidence suggests that stromal cells in the BM niche, including mesenchymal stem/stromal cells (MSCs), regulate EM pathways by sensing injury signals and relaying them to HSPCs and myeloid cells.

MSCs, originally identified as the major cellular constituents of the BM niche, play a crucial role in suppression of excessive immune response and maintenance of tissue homeostasis following injury primarily through modulation of myeloid cells 2-4. Importantly, MSCs are highly sensitive to microenvironmental perturbations, expressing a variety of pattern recognition receptors (PRRs), including Toll-like receptor (TLR) 3, TLR4 and nucleotide-binding oligomerization domain (NOD)1, that detect exogenous and endogenous danger signals produced during injury 5-7. Activation of these receptors impacts the actions of MSCs on hematopoiesis and myeloid cells 8-12 as well as MSCs' survival, differentiation, migration and immunoregulatory functions 5-7, 13-18.

Most studies thus far have focused on the implications of TLR3 and TLR4 for the immunoregulatory capacities of MSCs, yielding contradictory results 5-7, 19. It has been demonstrated that TLR3 and TLR4 activation elicit either immunosuppressive 20-22 or immunogenic phenotypes in MSCs 23-25. Alternatively, TLR3 or TLR4 activation in MSCs has been reported to exert differential effects on the immune system. TLR3 priming of MSCs boosted the production of immunosuppressive mediators, leading to inhibition of T cells and macrophages 13, 26-35, while TLR4 priming elaborated pro-inflammatory mediators, permitting T cell activation 13, 26, 36. These conflicting results reflect the ability of MSCs to dynamically adjust their responses to various microenvironmental conditions they encounter in the steady state or in injury.

The cornea is a transparent outer layer of the eye that allows transmission of light and contributes about two-thirds of refractive power. To serve its optical function, the cornea lacks both blood and lymphatic vessels, actively maintaining avascularity, which phenomenon is known as “corneal angiogenic privilege”. However, abnormal immune responses underlying many diseases of the cornea can disrupt angiogenic privilege and induce pathological corneal angiogenesis. In our previous studies, we observed that sterile injury to the cornea triggered the release of damage-associated molecular patterns (DAMPs) that activate TLR2, resulting in corneal inflammation, neovascularization (NV) and opacification 37, 38. Remarkably, MSCs suppressed inflammatory corneal NV and preserved corneal transparency by modulating monocytes/macrophages 39, 40.

Based on these findings, we hypothesized that the corneal microenvironment, being abundant in injury-derived TLR2 ligands, might affect the anti-inflammatory and anti-angiogenic effects of MSCs on the cornea during injury. To test this hypothesis, we herein examined the expression of TLR2 along with that of other TLRs in MSCs, and analyzed the functional significance of TLR2 activation using single-cell RNA sequencing (scRNA-seq). Additionally, we investigated the effects and mechanisms of TLR2 and its downstream activation on MSCs' immunoregulatory and angiostatic activities in monocytes/macrophages as well as in a mouse model of sterile injury-induced inflammatory corneal angiogenesis.

## Results

### MSCs upregulate functional TLR2 upon monocyte/macrophage coculturing

**Figure 1:**
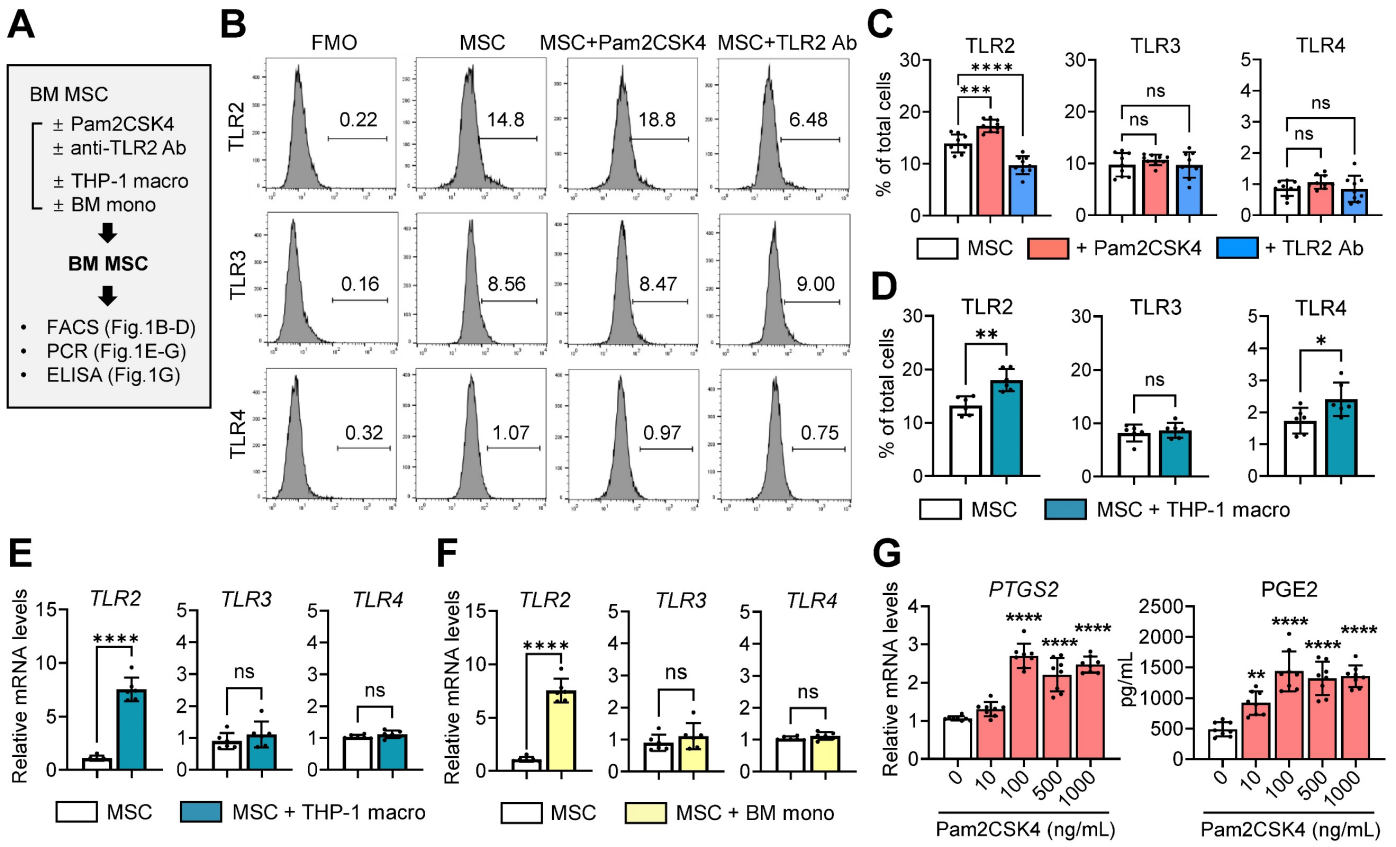
**Expression of TLRs in MSCs and effect of TLR2 stimulation on PTGS2/COX2-PGE2 pathway. A.** Experimental scheme. Human BM MSCs were treated with either Pam2CSK4 or anti-TLR2 Ab for 24 h. Alternatively, MSCs were cocultured in a Transwell system with PMA-differentiated THP-1 macrophages (THP-1 macro) for 18 h or with GM-CSF-stimulated murine BM monocytes (BM mono) for 5 d. Then, MSCs were assessed for expression of TLRs and *PTGS2* and production of PGE2. **B-D.** Representative and quantitative flow cytometry results for TLR2, TLR3 and TLR4 in MSCs. FMO (fluorescence minus one) control for each Ab was used as gating control. MSCs were treated with Pam2CSK4 (100 ng/mL) or anti-TLR2 Ab (10 μg/mL). **E, F.** qRT-PCR quantification for *TLR2*, *TLR3* and *TLR4* in MSCs cocultured with THP-1 macrophages or BM monocytes. The mRNA levels are presented as the fold changes relative to MSCs cultured alone. **G.** qRT-PCR for *PTGS2* and ELISA for PGE2 production in MSCs treated with Pam2CSK4 (0-1000 ng/mL). Shown are the mRNA levels relative to untreated MSCs. Data represent means ± SD from 2-3 independent experiments. *p < 0.05, **p < 0.01, ***p < 0.001, ****p < 0.0001, ns: not significant, as analyzed by one-way ANOVA and Tukey's test (**C, G**) or by Student's* t*-test (**D-F**).

Using flow cytometry, we evaluated the expression of TLR2, TLR3 and TLR4 in human BM-derived MSCs in the presence or absence of a TLR2 agonist Pam2CSK4 or an anti-TLR2 Ab (**Figure 1A**). The results revealed that MSCs expressed substantial levels of TLR2 and TLR3, whereas TLR4 expression was relatively low (**Figure 1B, C**). As expected, TLR2 expression was upregulated by Pam2CSK4 and downregulated by an anti-TLR2 Ab, whereas TLR3 and TLR4 expression remained unchanged by Pam2CSK4 or the anti-TLR2 Ab (**Figure 1B, C**).

Furthermore, we examined the effects of monocytes/macrophages on TLR expression profiles in MSCs (**Figure 1A**). Notably, coculturing with phorbol myristate acetate (PMA)-differentiated THP-1 macrophages or granulocyte-macrophage colony-stimulating factor (GM-CSF)-stimulated murine BM monocytes induced a significant increase in TLR2 expression in MSCs at both the mRNA and surface protein levels, whereas the effects on TLR3 or TLR4 levels were not significant (**Figure 1D-F**). Moreover, TLR2 stimulation of MSCs with Pam2CSK4 dose-dependently increased the levels of *PTGS2*, a gene encoding the enzyme prostaglandin endoperoxidase synthase 2/cyclooxygenase-2 (PTGS2/COX2), as well as the secretion of prostaglandin E2 (PGE2) synthesized by PTGS2/COX-2 (**Figure 1G**).

Together, the data indicate that monocytes/macrophages increase TLR2 in MSCs and that TLR2 stimulation activates the PTGS2/COX2-PGE2 pathway in MSCs.

### TLR2 in MSCs is important for monocyte/macrophage differentiation into suppressive phenotypes

Our group previously reported that MSCs drive the differentiation of monocytes/macrophages into anti-inflammatory phenotypes or myeloid-derived suppressor cells (MDSCs) largely through the PTGS2/COX2-PGE2 pathway 40, 41. Given the upregulation of the PTGS2/COX2-PGE2 pathway in TLR2-stimulated MSCs (**Figure 1G**), we next tested whether TLR2 activation in MSCs influences their actions on monocytes/macrophages.

**Figure 2:**
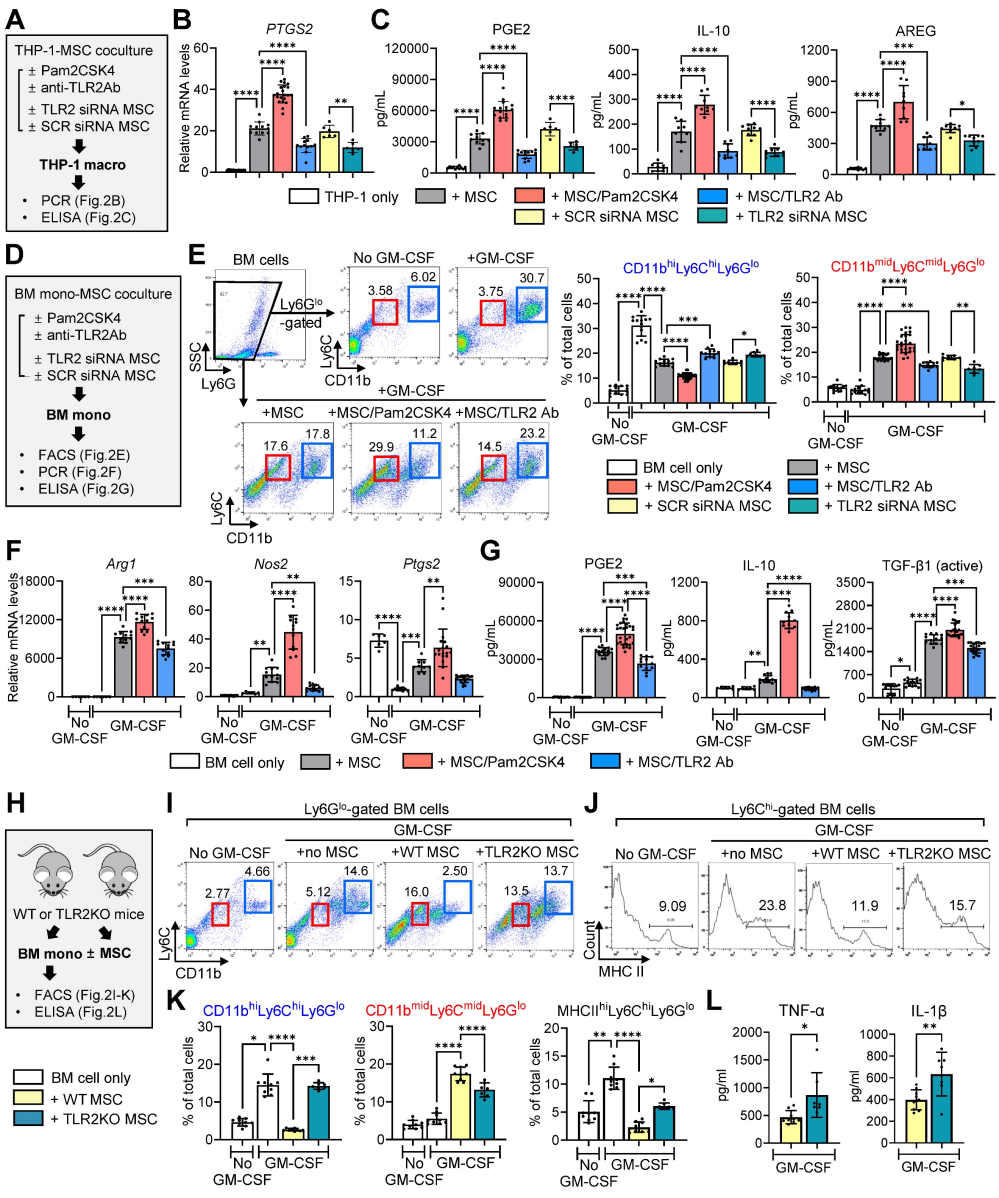
**TLR2 signaling in MSC is crucial to induction of immunosuppressive monocytes/macrophages. A.** Experimental scheme. THP-1 macrophages were cocultured with human MSCs in a Transwell system. Prior to coculturing, MSCs were subjected to pretreatment with Pam2CSK4 (100 ng/mL) or anti-TLR2 Ab (10 μg/mL) or transfected with TLR2 siRNA or SCR siRNA for 24 h. After 18 h of coculturing, THP-1 macrophages were analyzed for *PTGS2* mRNA levels and the secreted levels of PGE2, IL-10 and AREG. **B.** qRT-PCR for *PTGS2* in THP-1 macrophages*.* The mRNA levels are fold changes relative to THP-1 macrophages cultured alone. **C.** ELISA for PGE2, IL-10 and AREG in supernatants of THP-1 macrophages with or without MSC coculturing. **D.** Experimental scheme. Murine BM cells were cocultured with human MSCs under GM-CSF stimulation for 5 d. Prior to coculturing, MSCs were pretreated with Pam2CSK4 (100 ng/mL) or anti-TLR2 Ab (10 μg/mL) or transfected with TLR2 siRNA or SCR siRNA for 24 h. After 5 d of coculturing, BM cells were assayed. **E.** Representative and quantitative flow cytometry results for CD11b^hi^Ly6C^hi^Ly6G^lo^ and CD11b^mid^Ly6C^mid^Ly6G^lo^ cells in BM cells. **F.** qRT-PCR for *Arg1*, *Nos2* and* Ptgs2* in BM cells. Shown are the mRNA levels relative to BM cells cultured alone without GM-CSF. **G.** ELISA for PGE2, IL-10 and active TGF-β1 levels in cell-free supernatants of BM cells with or without MSC coculturing. Mouse-specific ELISA kits were used for measurement of IL-10 and active TGF-β1. The PGE2 assay kit recognizes both human and murine PGE2. **H.** Experimental scheme. MSCs were isolated from WT C57BL/6 or TLR2 KO mice. BM cells from WT C57BL/6 mice were cocultured with WT or TLR2 KO MSCs under GM-CSF for 5 d, and then subjected to assays. **I, J.** Representative flow cytometry results for CD11b, Ly6C, Ly6G and MHC class II in BM cells. **K.** Quantitative flow cytometric analysis for CD11b^hi^Ly6C^hi^Ly6G^lo^ cells, CD11b^mid^Ly6C^mid^Ly6G^lo^ cells and MHC class II^hi^Ly6C^hi^Ly6G^lo^ cells in BM cells. **L.** ELISA for murine TNF-α and IL-1β in supernatants of cell cultures. Data are means ± SD from 3 independent experiments. *p < 0.05, **p < 0.01, ***p < 0.001, ****p < 0.0001, as analyzed by one-way ANOVA and Tukey's test, Kruskal-Wallis test and Dunn's multiple-comparison test (CD11b^hi^Ly6C^hi^Ly6G^lo^ and MHC class II^hi^Ly6C^hi^Ly6G^lo^ cells in K) or Student's* t*-test (L).

We cocultured THP-1-differentiated macrophages in a Transwell system with MSCs that had been pretreated with Pam2CSK4 or an anti-TLR2 Ab, or transfected with scrambled (SCR) siRNA or TLR2 siRNA (**Figure 2A**). As was consistent with our previous observation 40, MSCs induced the secretion of PGE2, IL-10 and amphiregulin (AREG) in THP-1 macrophages as well as the levels of *PTGS2*, all of which are important mediators responsible for inflammation resolution and corneal regeneration (**Figure 2B, C**). TLR2 stimulation of MSCs with Pam2CSK4 markedly promoted these actions on THP-1 macrophages, whereas TLR2 blocking in MSCs with siRNA or Ab significantly abrogated them (**Figure 2B, C**).

In a parallel experiment, murine BM-derived monocytes were cocultured in a Transwell system with MSCs under GM-CSF stimulation (**Figure 2D**). In our previous studies, MSCs directed the differentiation of GM-CSF-stimulated BM cells from pro-inflammatory CD11b^hi^Ly6C^hi^Ly6G^lo^ monocytes into immunosuppressive CD11b^mid^Ly6C^mid^Ly6G^lo^ monocytic MDSCs 41, 42. Consistent with these findings, the present results revealed that MSCs generated a distinct population of CD11b^mid^Ly6C^mid^Ly6G^lo^ cells in BM monocytes, while reducing CD11b^hi^Ly6C^hi^Ly6G^lo^ cells (**Figure 2E**). Furthermore, MSCs enhanced the levels of MDSC markers (*Arg1* encoding arginase and *Nos2* encoding inducible nitric oxide synthase) and *Ptgs2* as well as the secretion of PGE2, IL-10 and active TGF-β1, which are major soluble factors mediating the immunosuppressive actions of MDSCs (**Figure 2F, G**). These effects of MSCs on MDSC differentiation and immunoregulatory factor production were markedly augmented by Pam2CSK4 treatment of MSCs and significantly attenuated by treatment of MSCs with anti-TLR2 Ab or TLR2 siRNA (**Figure 2E-G**).

Additionally, we isolated MSCs from wild-type (WT) C57BL/6 or TLR2 knockout (KO) mice and cocultured them with BM monocytes derived from WT C57BL/6 mice under GM-CSF stimulation (**Figure 2H**). MSCs from TLR2 KO mice were less effective in inducing CD11b^mid^Ly6C^mid^Ly6G^lo^ MDSCs and suppressing pro-inflammatory CD11b^hi^Ly6C^hi^Ly6G^lo^ monocytes and major histocompatibility complex (MHC) class II^hi^Ly6C^hi^Ly6G^lo^ monocytes compared to MSCs from WT C57BL/6 mice (**Figure 2I-K**). Moreover, TLR2 KO MSCs failed to inhibit the production of pro-inflammatory cytokines TNF-α and IL-1β in BM monocytes, relative to WT MSCs (**Figure 2L**).

Collectively, the results suggest that TLR2 signaling in MSCs is critical in order for MSCs to induce the differentiation and polarization of monocytes/macrophages from pro-inflammatory phenotypes into immunosuppressive phenotypes.

### TLR2 signaling in MSCs mediates their anti-inflammatory and angiostatic actions on the cornea

Next, we investigated the significance of TLR2 signaling in MSC activity *in vivo*. To this end, we employed a mouse model of suture-induced corneal NV, which is a well-established model of inflammatory corneal lymphangiogenesis and hemangiogenesis mediated by macrophage recruitment 43. Our group previously demonstrated the therapeutic effects of MSCs through modulation of macrophages in this model 39.

**Figure 3:**
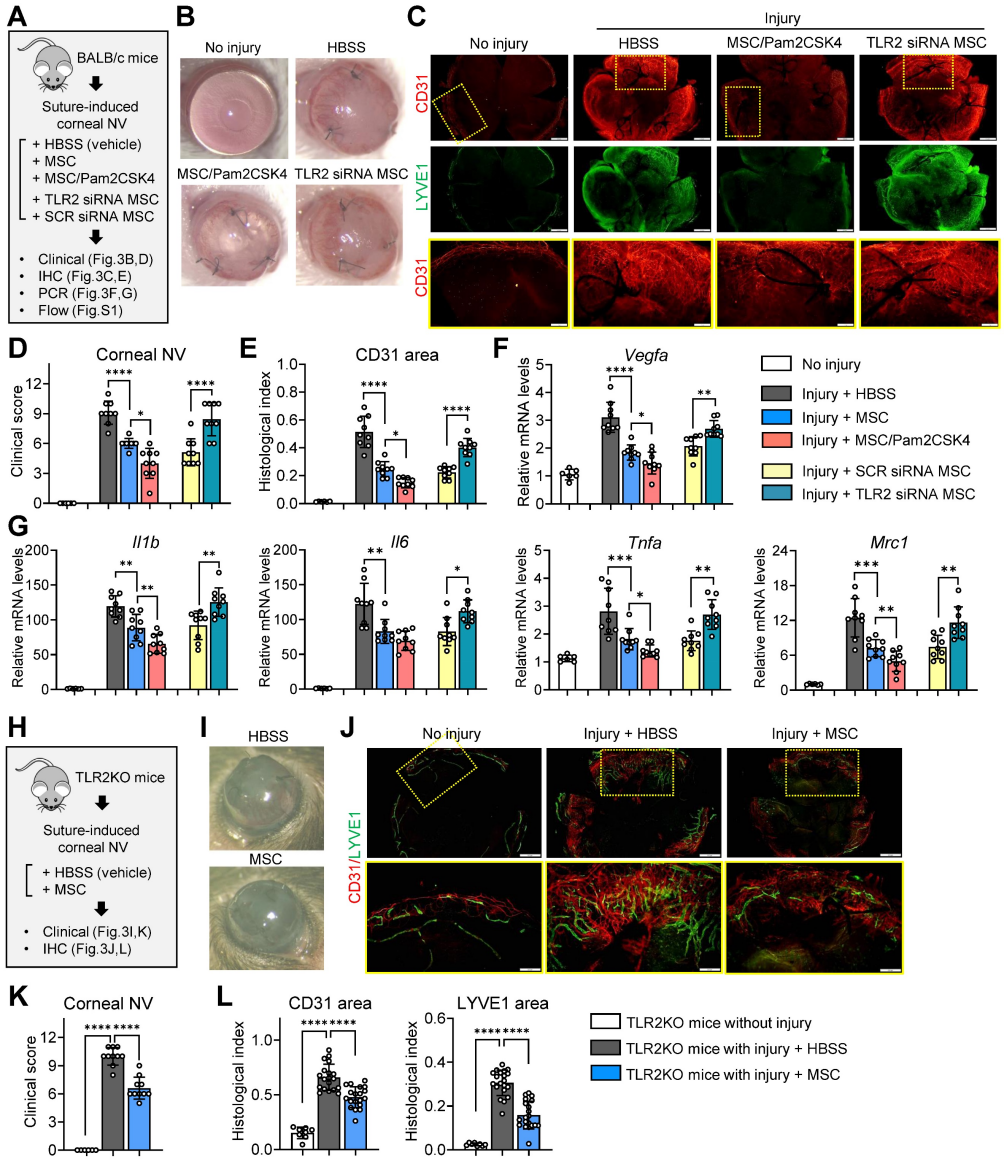
** TLR2 signaling in MSCs is essential for suppression of inflammatory angiogenesis in the cornea following sterile injury. A.** Experimental protocol. Immediately after corneal suturing injuries, MSCs, Pam2CSK4-pretreated MSCs, TLR2 siRNA-transfected MSCs, SCR siRNA-transfected MSCs or HBSS (vehicle) were administered via tail vein injection in BALB/c mice. Seven days later, the corneas were subjected to assays. **B, C.** Representative corneal photographs and microphotographs of CD31/LYVE1-stained corneal whole-mounts. Scale bar: 500 μm for the first and second rows and 200 μm for the third row (magnified images of yellow-outlined insets from the first row). **D, E**. Clinical scoring of corneal NV and quantification of CD31-stained area in corneal whole-mounts. **F, G.** qRT-PCR for pro-angiogenic factors and pro-inflammatory cytokines in cornea. The mRNA levels are presented relative to those in BALB/c control corneas that had not received injury or treatment. **H.** Experimental protocol. Corneal sutures were applied to TLR2 KO mice, and either MSCs or HBSS were intravenously injected. After 7 d, the corneas were subjected to assays. **I, J.** Representative corneal photographs and microphotographs after CD31/LYVE1 immunostaining. Scale bar: 500 μm for the upper row and 200 μm for the lower row (magnified images of yellow-outlined insets from the upper row). **K, L.** Clinical scoring of corneal NV and measurement of CD31- and LYVE1-stained areas. Data represent means ± SD, where a circle indicates the data from an individual animal. *p < 0.05, **p < 0.01, ***p < 0.001, ****p < 0.0001, as analyzed by one-way ANOVA and Tukey's test or by Student's* t*-test (Injury + MSC vs. Injury + MSC/Pam2CSK4 in F, G).

Following corneal suturing injury in BALB/c mice, we administered MSCs, Pam2CSK4-pretreated MSCs, TLR2 siRNA-transfected MSCs, SCR siRNA-transfected MSCs or the same volume of vehicle (Hank's balanced salt solution, HBSS) via tail vein injection. Seven days later, the eyes were analyzed (**Figure 3A**). In agreement with our previous findings 39, MSCs markedly inhibited corneal NV development as examined by slit-lamp biomicroscopy (**Figure 3B, D**) as well as corneal whole-mount immunostaining for a panendothelial marker CD31 and a lymphatic vessel marker LYVE1 (lymphatic vessel endothelial hyaluronan receptor-1) (**Figure 3C, E**). Moreover, MSCs significantly decreased the levels of pro-angiogenic and pro-inflammatory factors, including *Vegfa, Il1b, Il6, Tnfa* and *Mrc1*, in the cornea as well as the percentages of pro-inflammatory, pro-angiogenic CD11b^hi^Ly6C^hi^ monocytes in the spleen and blood (**Figure 3F, G, Figure S1**). Notably, pretreatment of MSCs with Pam2CSK4 enhanced the effects of MSCs on attenuating corneal NV and inflammation and suppressing pro-inflammatory monocytes, whereas TLR2 knockdown with siRNA abolished the MSC effects (**Figure 3B-G, Figure S1**).

In addition, we evaluated whether MSCs could elicit the therapeutic effects in TLR2 KO mice as they did in WT mice (**Figure 3H**). The results showed that systemic administration of MSCs was also effective in attenuating inflammatory corneal NV in TLR2 KO mice (**Figure 3I-L**), suggesting that TLR2 signaling in MSCs, not TLR2 signaling in recipients, is important for mediating the therapeutic effects of MSCs on the cornea.

Taken together, our findings demonstrate that TLR2 signaling in MSCs plays a crucial role in the cells' anti-inflammatory and angiostatic functions in the cornea as well as in monocytes/macrophages.

### TLR2 stimulation induces AKR1C1 in MSCs without altering cell phenotypes or clusters

**Figure 4:**
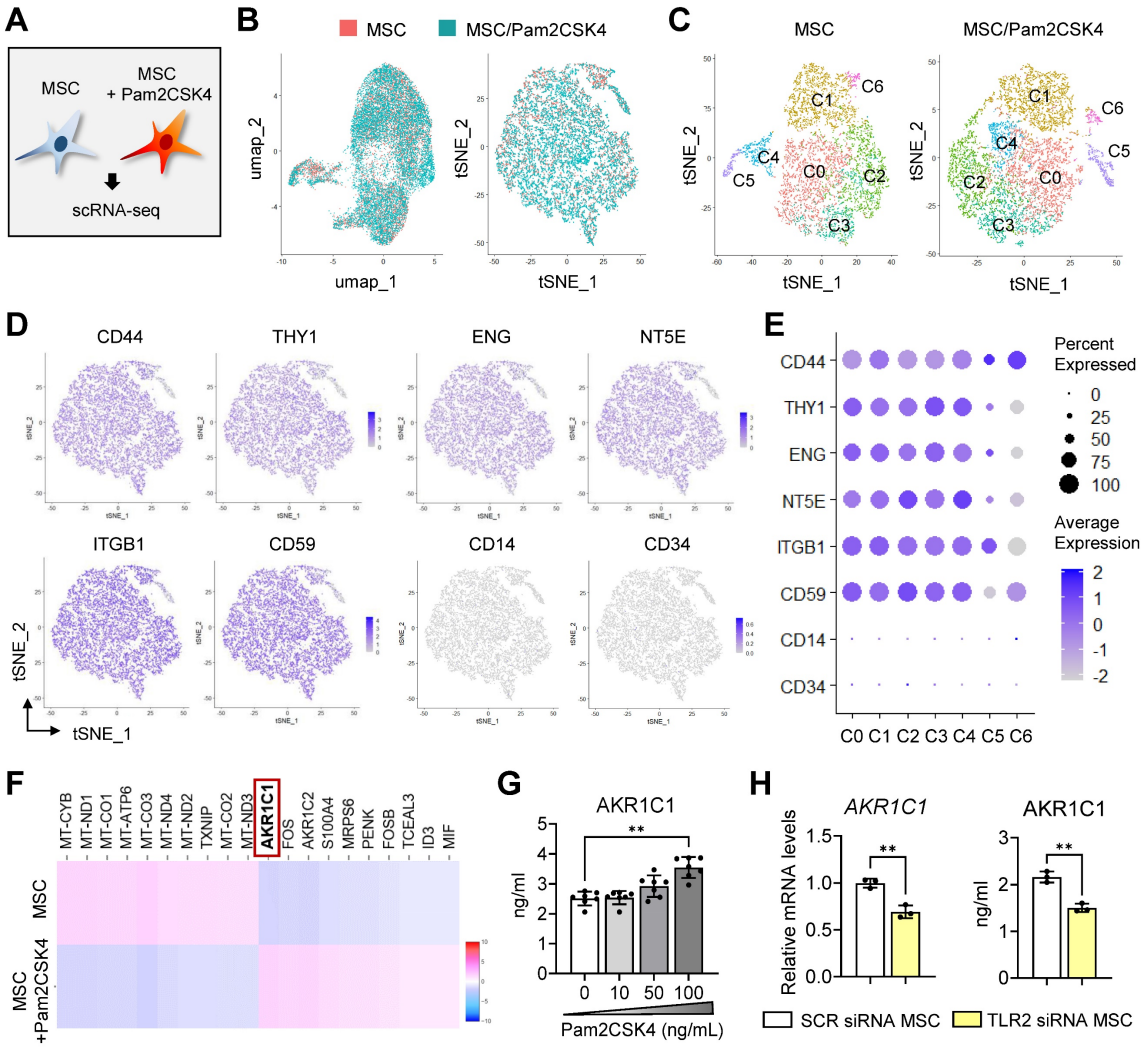
**Single-cell transcriptional profiling of Pam2CSK4-treated and untreated MSCs. A.** Experimental scheme. MSCs treated with Pam2CSK4 100 ng/mL for 24 h and untreated MSCs were subjected to scRNA-seq on the 10x Genomics Chromium platform. **B.** Combined UMAP and tSNE plots of Pam2CSK4-treated MSCs (blue) and untreated MSCs (orange) showing overlapping. **C.** tSNE plots of Pam2CSK4-treated MSCs and untreated MSCs depicting 7 clusters (C0-C6) in both cell libraries. **D, E.** Feature and dot plots displaying positive and negative markers for MSCs. **F.** Heatmap of top 10 DEGs in Pam2CSK4-treated MSCs vs. untreated MSCs as determined by RNA-Seq analysis. *AKR1C1* was identified as the top DEG upregulated in Pam2CSK4-treated MSCs relative to untreated MSCs. **G.** ELISA for secreted levels of AKR1C1 in cell-free supernatants of MSC cultures treated with Pam2CSK4 (0-100 ng/mL). **H.** qRT-PCR and ELISA for mRNA and protein levels of AKR1C1 in MSCs transfected with TLR2 siRNA or control SCR siRNA. Means ± SD are presented. **p < 0.01 as analyzed by Kruskal-Wallis test and Dunn's multiple-comparison test (G) or Student's* t*-test (H).

To elucidate the molecular signature of MSCs after TLR2 activation, we performed scRNA-seq on Pam2CSK4-treated and untreated MSCs using the 10x Genomics Chromium platform (**Figure 4A**).

The estimated number of cells analyzed and the total number of genes detected were 9,308 and 21,224 in Pam2CSK4-treated MSCs, respectively, and 6710 and 21,238 in untreated MSCs, respectively, as determined by the Cell Ranger count pipeline (10x Genomics). The median gene counts and UMI counts per cell were 3,483 and 11,976 in Pam2CSK4-treated MSCs, respectively, and 4,540 and 19,712 in untreated MSCs, respectively. The scRNA-seq data were deposited in the GEO databases (accession No. GSE273279; GSM8425610 for Pam2CSK4-treated MSCs and GSM8425609 for untreated MSCs).

The scRNA-seq data from the Pam2CSK4-treated and untreated MSCs were analyzed using Seurat. The combined analysis of the two cell libraries showed that Pam2CSK4 treatment did not induce any new cell clusters in MSCs, as evidenced by the overlap of the two cell datasets on both t-distributed stochastic neighbor embedding (t-SNE) and uniform manifold approximation and projection (UMAP) plots (**Figure 4B**). Unbiased, graph-based clustering of each cell library identified 7 clusters (C0 to C6) in both the Pam2CSK4-treated and untreated MSCs (**Figure 4C**). Positive markers for MSCs, such as CD44, CD90 (THY1), CD105 (endoglin, ENG), CD72 (5'-nucleotidase ecto, NT5E), CD59 and ITGB1 (integrin subunit beta 1), were robustly expressed across most clusters in both the Pam2CSK4-treated and untreated MSCs, while negative markers for MSCs, including CD14 and CD34, were not detectable in any clusters (**Figure 4D, E**).

Comparison of the two transcriptomes revealed that *AKR1C1*, a gene encoding aldo-keto reductase family 1 member C1, was the most significantly upregulated gene in the Pam2CSK4-treated MSCs compared with the untreated MSCs (**Figure 4F**). Indeed, Pam2CSK4 induced secretion of AKR1C1 in MSCs in a dose-dependent manner (**Figure 4G**). By contrast, both the mRNA and secreted protein levels of AKR1C1 were significantly repressed by TLR2 knockdown in MSCs (**Figure 4H**).

These results indicate that TLR2 activation in MSCs induces both transcription and secretion of AKR1C1 without altering cell phenotypes or affecting cell clusters comprising MSCs.

### AKR1C1 inhibits MSC ferroptosis

Next, we explored the functional significance of AKR1C1 in MSCs. Recent evidence has identified AKR1C1 as a ferroptosis-related gene/protein. Under oxidative stress, increased expression of AKR1C1 prevents ferroptosis by converting the end products of lipid peroxides into the corresponding nontoxic lipid-derived alcohols 44, 45. Building on this knowledge, we went on to evaluate the effects of AKR1C1 inhibition on MSCs' survival and susceptibility to ferroptosis.

**Figure 5:**
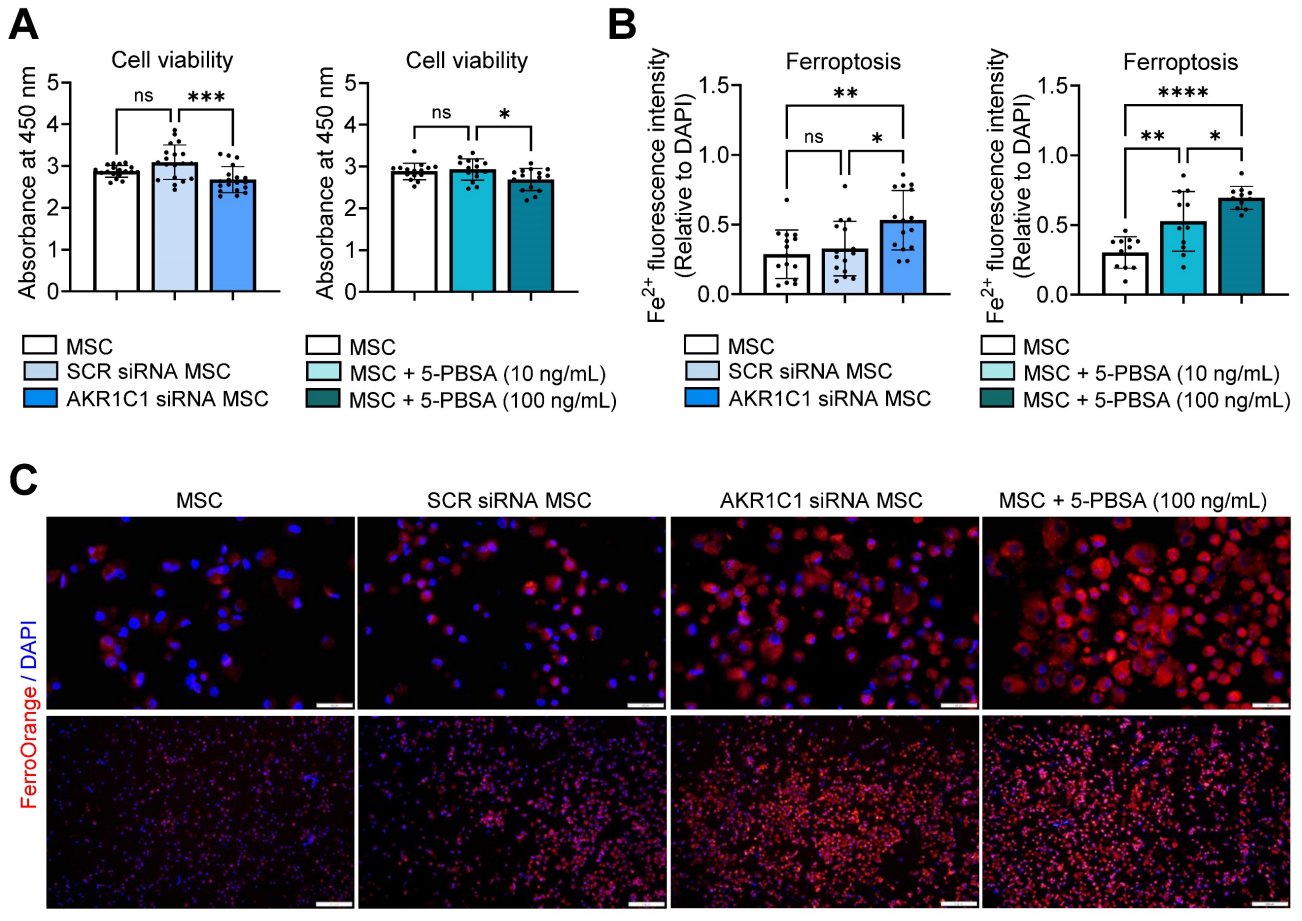
**AKR1C1 inhibition promotes MSC ferroptosis. A.** Cell viability assay in MSCs transfected with AKR1C1 siRNA or control SCR siRNA or in MSCs treated with AKR1C1 inhibitor, 5-PBSA (10 or 100 ng/mL). **B, C.** Quantification of intracellular Fe^2+^ fluorescence intensity in MSCs and representative fluorescence images. Red indicates FerroOrange staining, while blue represents DAPI-stained nuclei. Scale bar: 50 μm for the upper row and 200 μm for the lower row. Data are means ± SD from 3-4 independent experiments. *p < 0.05, **p < 0.01, ***p < 0.001, ****p < 0.0001, ns: not significant, as analyzed by one-way ANOVA and Tukey's test.

Knockdown of AKR1C1 in MSCs using siRNA transfection significantly reduced cell viability, as assessed by the Cell Counting Kit-8 (CCK-8) assay (**Figure 5A, Figure S2A**). Similar results were observed for MSCs treated with a specific inhibitor of AKR1C1, 5-PBSA (**Figure 5A, Figure S2B**). Additionally, staining of MSCs with the FerroOrange probe, a cell membrane-permeable dye, which enables live-cell fluorescent imaging of intracellular Fe^2+^, revealed a marked increase in ferroptosis in AKR1C1 siRNA-transfected MSCs relative to SCR siRNA-transfected MSCs or untreated MSCs (**Figure 5B, C**). Moreover, AKR1C1 inhibition with 5-PBSA dose-dependently increased ferroptosis in MSCs (**Figure 5B, C**).

Thus, our findings suggest that AKR1C1 protects MSCs against ferroptosis, thereby promoting the cells' survival.

### AKR1C1 regulates MSCs' therapeutic factor production and effects on monocytes/macrophages

**Figure 6:**
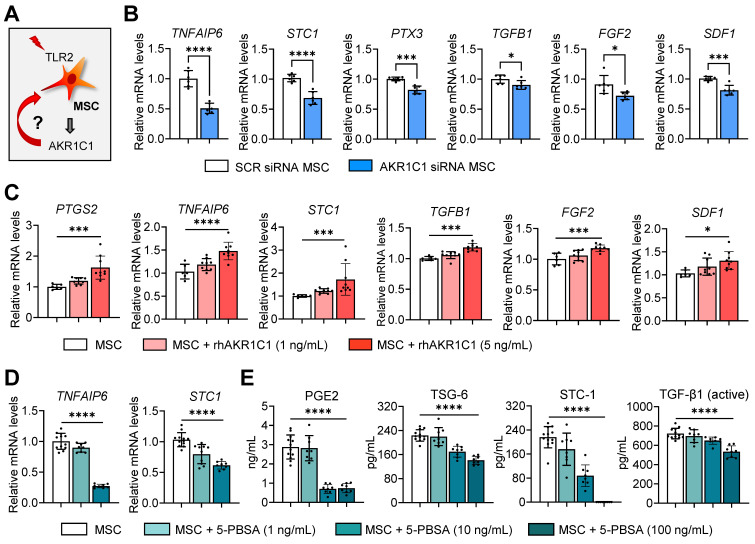
**AKR1C1 upregulates production of therapeutic factors in MSCs, whereas AKR1C1 inhibition abrogates it. A.** Experimental scheme. MSCs were subjected to AKR1C1 inhibition or replenishment and analyzed for the expression of key therapeutic factors. **B.** qRT-PCR for key therapeutic factors in MSCs after transfection with AKR1C1 siRNA or SCR siRNA. **C.** qRT-PCR in MSCs with rhAKR1C1 replenishment (1 or 5 ng/mL). **D, E.** qRT-PCR and ELISA for mRNA and secreted protein levels of key therapeutic factors in MSCs with 5-PBSA treatment (10 or 100 ng/mL). Data are means ± SD from 2-3 independent experiments. *p < 0.05, ***p < 0.001, ****p < 0.0001, as analyzed by one-way ANOVA and Tukey's test, Kruskal-Wallis test and Dunn's multiple-comparison test (*STC1*,* TGFB1*,* SDF1* in C and STC-1 in E) or Student's* t*-test (B).

In addition to MSC survival, we investigated the effects of AKR1C1 on the production of therapeutic factors by MSCs (**Figure 6A**). The mRNA levels of *TNFAIP6* (TNF-α induced gene/protein 6), *STC1* (stanniocalcin 1), *PTX3* (pentraxin 3), *TGFB1*, *FGF2* (fibroblast growth factor 2) and *SDF1* (stromal cell-derived factor 1), which are the key factors mediating MSCs' anti-inflammatory and angiostatic functions, were significantly downregulated in MSCs either by AKR1C1 knockdown or 5-PBSA treatment (**Figure 6B, D**). Conversely, supplementation of MSCs with recombinant AKR1C1 protein dose-dependently increased the expression of those factors (**Figure 6C**). Moreover, transcription and secretion of PGE2, TSG-6 (encoded by* TNFAIP6*), STC1 and TGF-β1 in MSCs were inhibited by 5-PBSA in a dose-dependent manner (**Figure 6D, E**).

It is well-established that MSCs modulate the activation of monocytes/macrophages largely through immunoregulatory factors, such as TSG-6, STC1 and PGE2 37, 40, 41, 46, 47. Therefore, we next evaluated the effects of AKR1C1 on MSC-mediated modulation of monocytes/macrophages. The results showed that MSCs with AKR1C1 knockdown were less effective than control MSCs in suppressing the generation of pro-inflammatory MHC class II^hi^Ly6C^hi^Ly6G^lo^ monocytes and the production of TNF-α and IL-6 in GM-CSF-stimulated BM monocytes (**Figure S3A-D**). Similar results were observed with THP-1-differenitated macrophages. The effects of MSCs on the suppression of the pro-inflammatory cytokine* IL-12A* and the induction of immunoregulatory proteins *PTGS2*/PGE2 and AREG in lipopolysaccharide (LPS)/adenosine triphosphate (ATP)-stimulated THP-1 macrophages were significantly lower with AKR1C1 siRNA-transfected MSCs compared to SCR siRNA-transfected MSCs (**Figure S3E-G**).

Together, the results suggest that AKR1C1 boosts the production of immunoregulatory factors in MSCs, thereby facilitating MSCs' regulatory actions on monocytes/macrophages.

### AKR1C1 directly affects monocyte/macrophage activation

AKR1C1 has been reported to alleviate inflammatory responses in an LPS-induced acute lung injury model, at least in part, through inhibiting overactivation of the JAK2/STAT3 signaling pathway 48. It is well-known that the STAT3 signaling pathway is involved in macrophage polarization 49. Thus, we next examined the direct effects of AKR1C1 on cultures of BM monocytes and THP-1 macrophages in our model.

**Figure 7:**
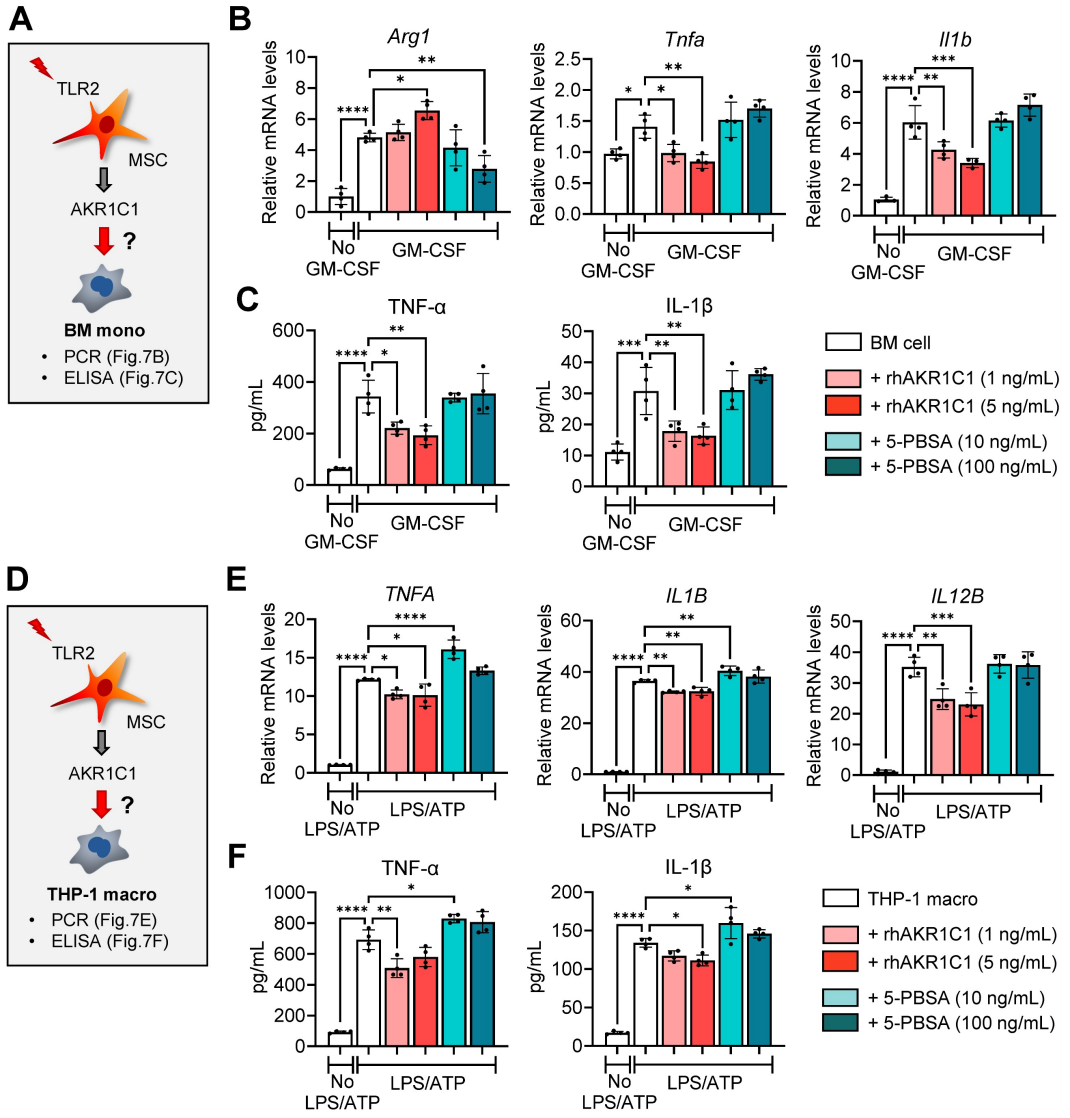
**AKR1C1 downregulates pro-inflammatory cytokines in monocytes and macrophages. A.** Experimental scheme. BM cells were cultured under GM-CSF stimulation for 5 d in the presence or absence of rhAKR1C1 (1 or 5 ng/mL) or 5-PBSA (10 or 100 ng/mL). Then, the cells were assessed for MDSC marker *Arg1* and pro-inflammatory cytokines. **B.** qRT-PCR for *Arg1*,* Tnfa* and *Il1b* in BM monocytes. The mRNA levels are presented as fold changes relative to GM-CSF-unstimulated BM cells. **C.** ELISA for TNF-α and IL-1β secretion in culture supernatants of BM monocytes. **D.** Experimental scheme. THP-1 macrophages were stimulated with LPS followed by ATP, and were treated with rhAKR1C1 (1 or 5 ng/mL) or 5-PBSA (10 or 100 ng/mL). 18 h later, the cells were analyzed for pro-inflammatory cytokine expression. **E, F.** qRT-PCR and ELISA for transcript and protein levels of TNF-α, IL-1β and IL-12B in LPS/ATP-stimulated THP-1 macrophages. Shown are the mRNA levels relative to LPS/ATP-unstimulated THP-1 macrophages. Means ± SD are presented. *p < 0.05, **p < 0.01, ***p < 0.001, ****p < 0.0001, as analyzed by one-way ANOVA and Tukey's test.

Addition of recombinant AKR1C1 dose-dependently increased the expression of the MDSC marker *Arg1*, while suppressing both transcription and secretion of pro-inflammatory cytokines TNF-α and IL-1β, in GM-CSF-stimulated BM monocytes (**Figure 7A-C**). Conversely, treatment with the AKR1C1 inhibitor 5-PBSA dose-dependently reduced *Arg1* expression in BM monocytes under GM-CSF stimulation, though it did not affect the secretion of pro-inflammatory cytokines (**Figure 7A-C**). Similar findings were observed with THP-1 macrophages stimulated with LPS and ATP. Supplementation with recombinant AKR1C1 significantly downregulated both the mRNA and protein levels of TNF-α and IL-1β as well as *IL12B* mRNA levels, whereas AKR1C1 inhibition using 5-PBSA upregulated the secreted levels of TNF-α and IL-1β in LPS/ATP-stimulated THP-1 macrophages (**Figure 7D-F**).

### AKR1C1 is required in order for MSCs to suppress inflammatory corneal NV

**Figure 8:**
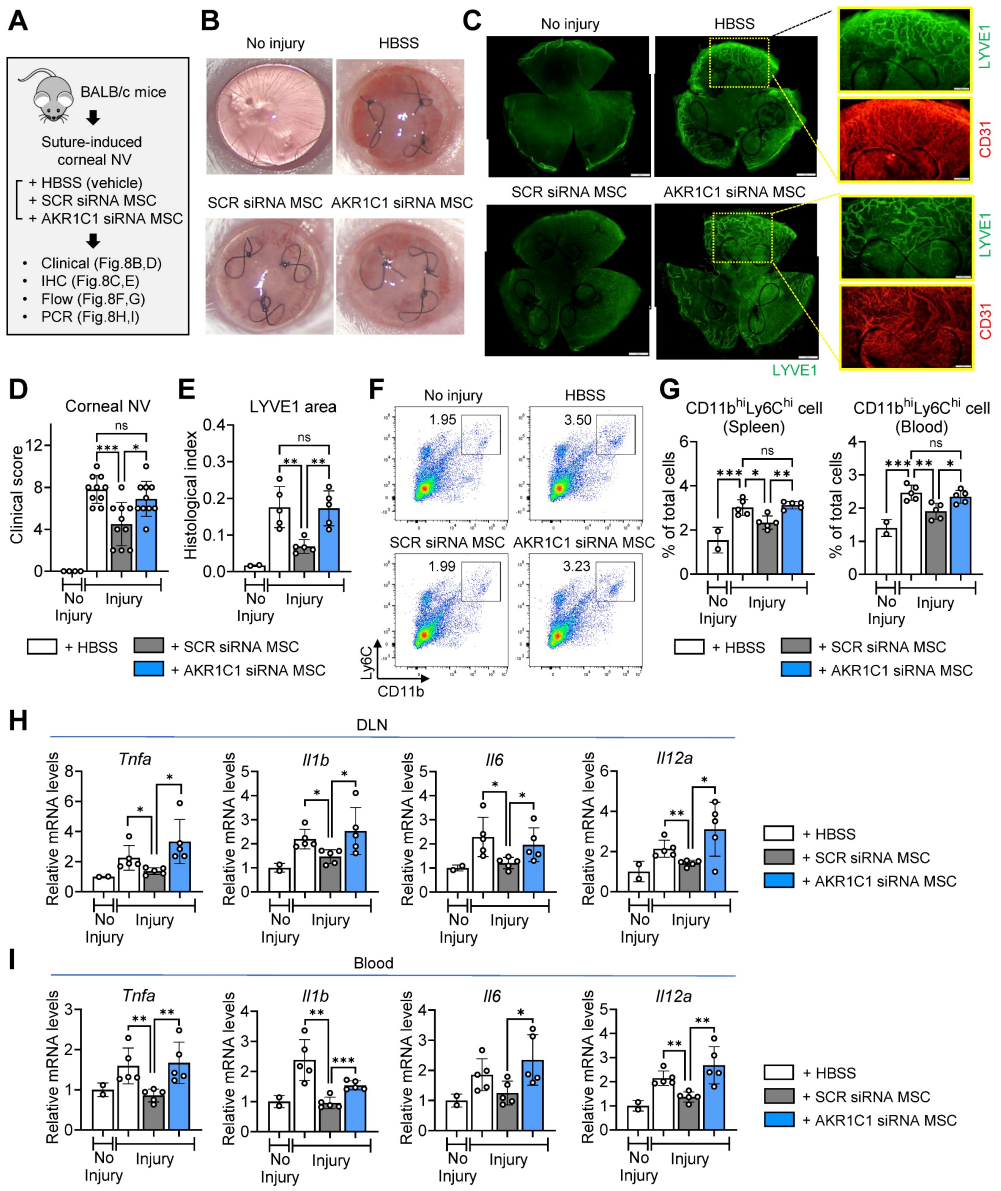
**AKR1C1 in MSCs is required for amelioration of inflammatory angiogenesis in the cornea following sterile injury. A.** Experimental protocol. Immediately after corneal suturing injury, AKR1C1 siRNA-transfected MSCs, SCR siRNA-transfected MSCs or HBSS (vehicle) were intravenously administered into BALB/c mice. Seven days later, the corneas were assayed. **B, C.** Representative corneal photographs and microphotographs of corneal flat mounts with CD31/LYVE1 immunostaining. Scale bar: 500 μm or 200 μm (magnified views of yellow-outlined insets). **D, E.** Clinical scoring of corneal NV and quantification of LYVE1-stained area in corneal whole-mounts. **F, G.** Representative flow cytometry cytograms of CD11b^hi^Ly6C^hi^ cells in the spleen (F) and quantitation of CD11b^hi^Ly6C^hi^ cells in spleen and blood (G). **H, I.** qRT-PCR assays for pro-inflammatory cytokines in ocular draining cervical lymph nodes (DLNs) and blood cells. The mRNA levels are presented as fold changes relative to naïve BALB/c mice without injury or treatment. Data represent means ± SD, where a circle indicates the data from an individual animal. *p < 0.05, **p < 0.01, ***p < 0.001, ns: not significant, as analyzed by one-way ANOVA and Tukey's test.

Finally, to confirm the role of AKR1C1 in the actions of MSCs *in vivo*, we intravenously infused AKR1C1 siRNA-transfected MSCs, SCR siRNA-transfected MSCs or HBSS (vehicle) into mice following suturing injury to the cornea in BALB/c mice (**Figure 8A**).

Whereas corneal NV was significantly decreased in the mice receiving MSCs with SCR siRNA, there was no significant reduction in corneal NV in the mice receiving MSCs with AKR1C1 siRNA, relative to those receiving HBSS (**Figure 8B-E**). Also, the percentages of pro-inflammatory, pro-angiogenic monocytes in the spleen and blood were significantly lowered by SCR siRNA-transfected MSCs but not by AKR1C1 siRNA-transfected MSCs (**Figure 8F, G**). Similar results were observed for the levels of pro-inflammatory cytokines, including *Tnfa*, *Il1b*, *Il6* and *Il12a*, in ocular draining lymph nodes and blood (**Figure 8H, I**).

Collectively, these data indicate that AKR1C1 is essential in order for MSCs to exert their anti-inflammatory and anti-angiogenic actions on the cornea.

## Discussion

Our results demonstrate that MSCs are activated by monocytes/macrophages to upregulate TLR2, and that TLR2 activation on MSCs induces expression of AKR1C1. AKR1C1 protects MSCs from ferroptosis, increases secretion of therapeutic factors, enhances MSCs' actions in driving differentiation of monocytes/macrophages into immunosuppressive phenotypes, and promotes MSCs' functions in suppressing pathologic inflammatory corneal NV (as summarized in **Graphical abstract**). These findings provide mechanistic insights into the therapeutic effects of MSCs on the cornea. In the corneal microenvironment where TLR2 ligands act as major DAMPs during sterile injury 37, 38, TLR2 activation in MSCs, leading to AKR1C1 secretion, plays a crucial role in the immunoregulatory and angiostatic effects of MSCs on corneal inflammation and pathologic NV.

In response to injury, MSCs are activated to regulate the immune system and suppress excessive immune responses, ultimately leading to tissue homeostasis. Sensing of injury signals through PRRs, including the TLR and NOD families, is an important step in MSCs' initiation of such functions. Previous studies have demonstrated that the responses of MSCs are largely heterogeneous depending on the types of ligands and activation of their corresponding receptors. For example, an elegant study by Shi et al. demonstrated that MSCs in the BM were activated by circulating TLR4 ligands in the bloodstream to rapidly produce monocyte chemotactic protein-1 and induce emigration of inflammatory monocytes from BM to fight against bacterial infection 10. A study by Iwamura et al. showed that MSCs sensed commensal microbiota via NOD1, leading to production of multiple hematopoietic cytokines such as IL-7, Flt3L, stem cell factor, thrombopoietin or IL-6 and thereby maintaining steady-state hematopoiesis 9. Apart from their role in BM, TLR activations in MSCs have been demonstrated to be directly associated with immunosuppressive functions and therapeutic efficacies of the cells in diseases. For example, TLR3-activated MSCs exhibited increased anti-inflammatory effects on macrophages, resulting in improved therapeutic outcomes in mice with cecal ligation and puncture-induced sepsis 28 or taurocholate-induced acute pancreatitis 27. TLR4-deficient MSCs displayed impaired capacity to inhibit Th1 and Th17 cell differentiation compared with WT MSCs, and thus lost their ability to ameliorate experimental autoimmune encephalomyelitis 19. By contrast, MSCs from TLR4 KO mice were shown to confer enhanced cardioprotection in rats with myocardial ischemia-reperfusion (IR) injury by increased production of angiogenic factors 50. This functional heterogeneity of MSCs not only is reflected by a variety of receptors MSCs express, but also accounts for MSCs' well-known efficacies in treating a broad spectrum of injuries in diverse tissues.

In this study, we focused on the implication of TLR2 activation in the actions of MSCs on monocytes/macrophages and the cornea, based on our two observations. First, our decades-long research has indicated that TLR2 ligands are major DAMPs in the cornea upon sterile injury 37, 38, and that MSCs exert dramatic therapeutic effects on the cornea primarily by modulating monocytes/macrophages 39, 40, 51, 52. Second, in this study, we observed that the levels of TLR2 in MSCs were upregulated by coculturing with monocytes/macrophages. Indeed, our findings indicated that TLR2 activation is crucial in order for MSCs to suppress pro-inflammatory monocytes/macrophages, induce suppressive macrophages and MDSCs, and exert anti-angiogenic and anti-inflammatory effects on the injured cornea. In line with our findings, Yu and Chiang reported that TLR2-stimulated MSCs alleviated airway inflammation in mice with ovalbumin-induced asthma more effectively than did control MSCs 53. Similarly, Abarbanell et al. found that TLR2 KO MSCs, unlike WT MSCs, did not improve functional myocardial recovery in mice following acute IR injury 54.

Our data also provide mechanistic insights into how TLR2 ligation licenses the anti-inflammatory and immunomodulatory activities of MSCs. Activation of TLR2 signaling in MSCs led to the upregulation of multiple therapeutic factors that MSCs secrete in response to injury, without affecting the cells' phenotype or cell compositions, as examined by scRNA-seq. Chief among the most highly-upregulated factors was AKR1C1, which was found to be primarily responsible for the induction of key therapeutic factors in MSCs. AKR1C1 belongs to the aldo-keto reductase (AKR) protein superfamily, which catalyzes the reduction of carbonyl substrates 55. Accumulating evidence demonstrates that the AKR1C subfamily, containing the four isoforms AKR1C1 to AKR1C4, plays a critical role in the regulation of various biological processes through both catalytic-dependent and -independent pathways. While most studies on AKR1Cs thus far have been performed in the context of tumors due to their abundant expression in various tumor tissues 56, recent research has expanded into non-tumor microenvironments. For instance, in an LPS-induced acute lung injury model, AKR1C1 overexpression reduced oxidative stress, attenuated inflammation, and improved tissue repair through deactivation of the JAK2/STAT3 pathway, whereas AKR1C1 KO resulted in increased lung injury 48. In a dry eye disease model, AKR1C1 was upregulated in corneal epithelial cells under desiccating stress to protect corneal epithelial cells against ferroptosis-induced damage and attenuate inflammation on the ocular surface 45. In addition to these reports, our study provides further evidence on the role of AKR1C1 as a negative regulator of inflammation. Specifically, AKR1C1 augmented the anti-inflammatory activities of MSCs by stimulating the production of immunoregulatory factors and inhibiting MSC ferroptosis, leading to improved corneal protection under injury. Moreover, AKR1C1 directly downregulated the pro-inflammatory activation of monocytes/macrophages. Although we did not investigate the direct effects of MSC-derived AKR1C1 on corneal epithelial cells, it is possible that AKR1C1 secreted from MSCs also protected the cornea by preserving corneal epithelial cells against damage, as indicated by the research by Zuo et al 45. Further investigation is warranted to elucidate the signaling pathways through which AKR1C1 induces therapeutic factors in MSCs and suppresses pro-inflammatory factors in monocytes/macrophages. One such candidate pathway is JAK/STAT3 signaling 48, 49.

MSCs are among the most extensively tested cell types in clinical trials. The safety profile of MSC therapy has proven excellent. However, the therapeutic efficacy is variable largely due to MSCs' inherent functional heterogeneity and complexity. One of the strategies to address these issues is to tailor MSCs for a particular disease indication based on their specific mechanism of action in that disease, thereby maximizing therapeutic potency prior to infusion into patients. Our study suggests that targeting the TLR2-AKR1C1 axis in MSCs could be a promising approach to prime and potentiate MSCs before clinical use in patients with corneal diseases. Future studies are needed to select the optimal methods for such manipulations, including genetic engineering of MSCs to overexpress AKR1C1 or chemical/pharmacological preconditioning for TLR2 stimulation.

The most salient feature of MSCs is the ability to interact with their microenvironment and neighboring cells, which allows MSCs to promptly recognize tissue perturbations and induce specific responses to restore and maintain homeostasis in the injured tissue. Specifically, in the cornea, MSCs detect TLR2 ligands generated during injury and are activated to secrete AKR1C1. Subsequently, AKR1C1 stimulates the production of a cascade of therapeutic mediators from MSCs, which contribute to corneal immunologic and angiogenic privilege and also protects MSCs and corneal cells against oxidative stress. These findings not only advance our understanding of the mechanisms underlying the homeostatic actions of MSCs but also provide a potential target for MSC priming to improve therapeutic efficacy in corneal diseases.

## Methods

### Cells and reagents

Primary human BM-derived MSCs were obtained from the Center for the Preparation and Distribution of Adult Stem Cells at the Institute for Regenerative Medicine, Texas A&M University, which provided standardized preparations of MSCs enriched for early progenitor cells under the auspices of an NIH/NCRR grant (P40 RR 17447-06). Passage 2 MSCs were expanded in complete culture media (CCM) composed of α-MEM (Gibco/Thermo Fisher Scientific, Waltham, MA) supplemented with 17% (vol/vol) fetal bovine serum (FBS; Gibco), 1% penicillin-streptomycin (PS; Lonza, Walkersville, MD) and 2 mM L-glutamine (Lonza) at 37°C in a 5% CO_2_ incubator.

For TLR2 activation or blocking, MSCs were treated with 10-1000 ng/mL Pam2CSK4 (tlrl-pm2s-1, InvivoGen, San Diego, CA) or 10 μg/mL anti-human TLR2 Ab (pab-hstlr2, InvivoGen) for 24 h. For TLR2 or AKR1C1 gene knockdown, MSCs were transfected with 10 μM TLR2 siRNA (sc-40256, Santa Cruz Biotechnology, Dallas, TX), the corresponding SCR siRNA (sc-37007, Santa Cruz Biotechnology), 10 μM AKR1C1 siRNA (AM16708, Ambion, Calsbad, CA) or the corresponding SCR siRNAs (AM4611, Ambion) using Lipofectamine^TM^ RNAiMAX transfection reagent (Invitrogen/Thermo Fisher Scientific, Waltham, MA). The knockdown efficiencies were confirmed to be > 90% at 18 h after the start of transfection. For AKR1C1 inhibition, the cells were treated with 1-100 ng/mL 5-PBSA (EAA90668, Biosynth, Staad, Switzerland). For AKR1C1 supplementation, recombinant human (rh) AKR1C1 (6529-DH-020, R&D Systems, Minneapolis, MN) was added to cultures at concentrations of 1-5 ng/mL.

Primary murine BM-derived MSCs were prepared by flushing the femurs and tibias of WT C57BL/6 or TLR2 KO mice using a 21-23G needle attached to a plastic syringe containing CCM and filtering them through a 70-μm cell strainer (Corning, Corning, NY). After centrifugation at 300 g for 10 min, the resultant suspensions were treated with ACK Lysing Buffer (Gibco) for the lysis of red blood cells (RBCs) and then cultured in CCM and the EasySep™ Mouse Mesenchymal Stem/Progenitor Cell Enrichment Kit (19771, STEMCELL Technologies, Vancouver, Canada). Subsequently, the cells were plated at a density of 5000 cells/cm^2^ and cultured in MesenCult™ Mesenchymal Stromal Cell Culture (05513, STEMCELL Technologies). The media were changed every 2 d or 3 d. The cells were confirmed negative for CD11b and CD31 and positive for CD29 and CD44 as examined by flow cytometry.

Primary BM monocytes were isolated by flushing the femurs of WT C57BL/6 mice using a 30G needle and a plastic syringe and filtering them through a 70-μm cell strainer (Corning). After RBC cell lysis in ACK Lysing Buffer (Gibco), the BM cells were cultured in DMEM (Welgene, Daegu, Korea) supplemented with 10% (vol/vol) heat-inactivated FBS (Gibco) and 1% PS (Lonza) at 37°C in 5% CO_2_. For pro-inflammatory monocyte differentiation, the cells were treated with GM-CSF (40 ng/mL, GenScript, Piscataway, NJ) for 5 d 41, 42.

THP-1 cells were obtained from a cell bank in Seoul National University Hospital (Seoul, Korea) and cultured in RPMI1640 media (Welgene) with 2% (vol/vol) heat-inactivated FBS (Gibco) and 1% PS (Lonza) at 37°C in 5% CO_2_. THP-1 cells were differentiated into macrophages by treatment with 300 ng/ml PMA (Sigma-Aldrich, St. Louis, MO) for 3 h. Twenty-four h later, THP-1-differentiated macrophages were stimulated with 2 μg/mL LPS (InvivoGen) for 3 h followed by 5 mM ATP (InvivoGen) for 45 min 40, 47.

For coculture studies, MSCs were plated in Transwell inserts and cultured in CCM with or without treatment with Pam2CSK4, anti-TLR2 Ab, TLR2 siRNA, AKR1C1 siRNA or SCR siRNA for 24 h. Then, the MSCs were cocultured with BM monocytes for 5 d or with THP-1-differentiated macrophages for 18 h at an MSCs to monocytes/macrophages ratio of 1:5.

### Mice and animal model

All of the animal procedures were approved by the Institutional Animal Care and Use Committee of Seoul National University Hospital Biomedical Research Institute (Seoul, Korea) and were performed in strict accordance with the ARVO (Association for Research in Vision and Ophthalmology) statement on the use of animals in ophthalmic and vision research.

BALB/c and C57BL/6 mice were purchased from KOATECH (Pyeongtaek, Korea), and TLR2 KO mice were obtained from Jackson Laboratory (Bar Harbor, ME). Experiments were conducted using 8-week-old mice.

To establish a suture-induced inflammatory corneal NV model, mice were anesthetized by intraperitoneal injection of zolazepam-tiletamine (Zoletil^®^, Virbac, Carros, France) and topical administration of 0.5% proparacaine hydrochloride ophthalmic solution (Hanmi Pharm Co., Ltd., Seoul, Korea). Three 10-0 nylon sutures were placed into the corneal stroma 2 mm from the pupil center and 120° apart from each other, with the knots left unburied 39, 57. Immediately after suture placement, cells (1 x 10^6^ cells in 100 μL HBSS per mouse) or the same volume of vehicle (HBSS) were administered to the mice via tail vein injection. Seven days later, the corneas and tissues were assayed.

### scRNA-seq

MSCs were treated with 100 ng/mL Pam2CSK4 (InvivoGen) for 24 h. Pam2CSK4-treated MSCs and untreated MSCs were subjected to scRNA-seq. The 10x Genomics Chromium platform was used to capture and barcode the cells, generating single-cell Gel Beads-in-Emulsion (GEMs) following the manufacturer's protocol. Briefly, cell suspensions were loaded with the reverse transcription master mix onto 10x Genomics Single Cell Chip G, where cells were partitioned into the GEMs along with gel beads coated with oligonucleotides. These oligonucleotides facilitated mRNA capture inside the droplets by 30 bp oligo-dT after cell lysis and provided barcodes to index cells (16 bp) as well as transcripts (12 bp UMI). Following reverse transcription, cDNAs with both barcodes were amplified, and libraries were compiled using the Single Cell 3' Reagent Kit for each sample. The libraries were then sequenced on an Illumina NovaSeq 6000 System (Illumina, San Diego, CA) in the 2 × 150 bp paired-end mode.

Sample demultiplexing, barcode processing and UMI counting were performed using the 10x Genomics pipeline Cell Ranger (https://support.10xgenomics.com). Raw base call files generated by Illumina sequencers were demultiplexed into reads in FASTQ format using the bcl2fastq software developed by Illumina (https://github.com/brwnj/bcl2fastq). The raw reads were trimmed from the 3' end to obtain the recommended number of cycles for read pairs (Read1: 28 bp; Read2: 90 bp). The reads of each library were then processed separately using the “cellranger count” pipeline to generate a gene-barcode matrix for each library. During this step, the reads were aligned to a human reference genome (version: hg19), and cell barcodes and UMIs associated with the aligned reads were corrected and filtered. scRNA-seq expression data were analyzed with Seurat for PCA, clustering, t-SNE and UMAP.

### qRT-PCR

Corneal tissues or cells were lysed in RNA isolation reagent (RNA Bee, Tel-Test Inc., Friendswood, TX) and homogenized with an ultrasound sonicator (Ultrasonic Processor, Cole Parmer Instruments, Vernon Hills, IL). Total RNA was extracted using the RNeasy Mini kit (Qiagen, Valencia, CA) and converted to cDNA by reverse transcription using the High Capacity RNA-to-cDNA^TM^ Kit (Applied Biosystems, Carlsbad, CA). Real-time amplification for specific molecules was performed using human- or mouse-specific TaqMan^®^ probes (TaqMan^®^ Gene Expression Assay kits, Applied Biosystems) and TaqMan^®^ Universal PCR Master Mix (Applied Biosystems) and analyzed on the ABI 7500 Real Time PCR System (Applied Biosystems). Data were normalized to *GAPDH* or *Gapdh*, analyzed by the 2^-∆∆Ct^ method, and presented as fold changes of mRNA levels relative to controls*.*

### ELISA

The cell-free supernatants were assayed for the levels of PGE2 (KGE004B, Prostaglandin E2 Parameter Assay Kit, R&D Systems), human AREG (DY262, R&D Systems), human IL-10 (DY217, R&D Systems), human active TGF-β1 (DY240, R&D Systems), human AKR1C1 (MBS7218140, Mybiosource, SanDiego, CA), human TSG-6 (LS-F50058, LSBio, Lynnwood, WA), human STC-1 (DY2958, R&D Systems), human TNF-α (DY210, R&D Systems), human IL-1β (DY201, R&D Systems), mouse IL-10 (DY417, R&D Systems), mouse active TGF-β1 (DY1679, R&D Systems), mouse TNF-α (DY410, R&D Systems) or mouse IL-1β (DY401, R&D Systems) according to the manufacturer's instructions.

### Flow cytometry

MSCs were stained with fluorescence-conjugated Abs against TLR2 (11-9922, Invitrogen), TLR3 (12-9039, Invitrogen) or TLR4 (17-9917, Invitrogen). BM monocytes were stained with Abs against CD11b (25-0112, eBioscience/Thermo Fisher Scientific, Waltham, MA), Ly6C (45-5932, eBioscience), Ly6G (11-5931, eBioscience) or MHC class II (17-5321, eBioscience).

For flow cytometric analysis of spleen and blood, the spleen was minced using the frosted ends of two glass slides in RPMI1640 medium (Welgene) containing 10% FBS (Gibco) and filtered through a 70-μm cell strainer (Corning). The resultant suspension was centrifuged, and single cell suspension of the spleen was acquired. Blood cells were collected by treating whole blood with ACK Lysing Buffer (Gibco) and centrifugation. Spleen and blood cells were stained with anti-CD11b Ab (25-0112, eBioscience) and anti-Ly6C Ab (45-5932, eBioscience).

### Corneal NV quantification

Corneal NV was quantified using both clinical scoring and CD31/LYVE1 co-immunostaining of whole-mount corneas.

For clinical scoring of corneal NV, the eyes were examined under a slit-lamp biomicroscope and photographed with a camera mounted on a surgical operating microscope. The amount of corneal NV was quantified on a scale of 0 to 3 in each quadrant of the cornea, yielding a total score of 0 to 12, based on the standardized scaling system: score 0: no new vessels; score 1: new vessels at the corneal limbus; score 2: new vessels spanning the limbus and approaching the corneal center; score 3: new vessels spanning the center 39, 57.

For CD31/LYVE1 staining, whole-mount corneas were fixed with a mixture of acetone and methanol (1:1) for 10 min at room temperature (RT). After 2% bovine serum albumin blocking for 1 h at RT, the tissues were immunostained with anti-CD31 Ab (1:20) (BD Pharmingen, San Diego, CA) or anti-LYVE1 Ab (1:100) (AngioBio, San Diego, CA) overnight at 4°C, followed by incubation with goat anti rat IgG-cy3 (Invitrogen) for 3 h at RT. After washing with phosphate-buffered saline, the stained tissues were flat-mounted onto glass slides and observed under a fluorescence confocal microscope (Olympus, Tokyo, Japan). For quantification of the CD31- or LYVE1-stained area, digital fluorescence images of corneal flat mounts were analyzed using ImageJ software (National Institutes of Health, Bethesda, MD) 58. Briefly, the total cornea was manually delineated by outlining the innermost vessels of the limbal arcade. Blood and lymphatic vessel sprouts were connected on the inner side, and corneal NV was defined as the area between the outline of the total cornea and the inner line of vessel sprouts. The ratio of the corneal NV area relative to the total corneal area was calculated and presented as a histologic index.

### Cell viability assay

Cell viability was quantified using the CCK-8 assay (CK04, DOJINDO Laboratories, Kumamoto, Japan) according to the manufacturer's instructions.

### Intracellular Fe^2+^ measurement

For detection of intracellular ferrous ion (Fe^2+^), MSCs were treated with 1 μM FerroOrange (F374, DOJINDO Laboratories) for 30 min in a 5% CO_2_ incubator at 37°C and observed under a fluorescence microscope (Olympus). The fluorescence intensity was quantified with ImageJ software (National Institutes of Health, Bethesda, MD).

### Statistical analysis

Statistical tests were conducted using Prism software (GraphPad, San Diego, CA). The normal distribution of data was assessed using the D'Agostino & Pearson test, Shapiro-Wilk test or Kolmogorov-Smirnov test. For comparisons among more than two groups, a one-way ANOVA with Tukey's test or the Kruskal-Wallis test with Dunn's multiple-comparisons test was applied. For comparisons between two groups, the Student's *t*-test or Mann-Whitney *U* test was used. All data are presented as the mean ± SD with statistical significance set at *p* < 0.05.

## Supplementary Material

Supplementary figures.
