# Supplementary material for: Activation of Toll-like receptor 2 promotes mesenchymal stem/stromal cell-mediated immunoregulation and angiostasis through AKR1C1
Source: Theranostics. 2024 Aug 6;14(12):4713–29. doi: 10.7150/thno.100327 (PMC11373616; doi:10.7150/thno.100327)
Supplement: Supplementary file 1 — Supplementary figures. [file thnov14p4713s1.pdf]

Supplementary figures and figure legends

Figure S1. Effect of TLR2 signaling in MSCs on the levels of pro-inflammatory monocytes in mice after corneal injury

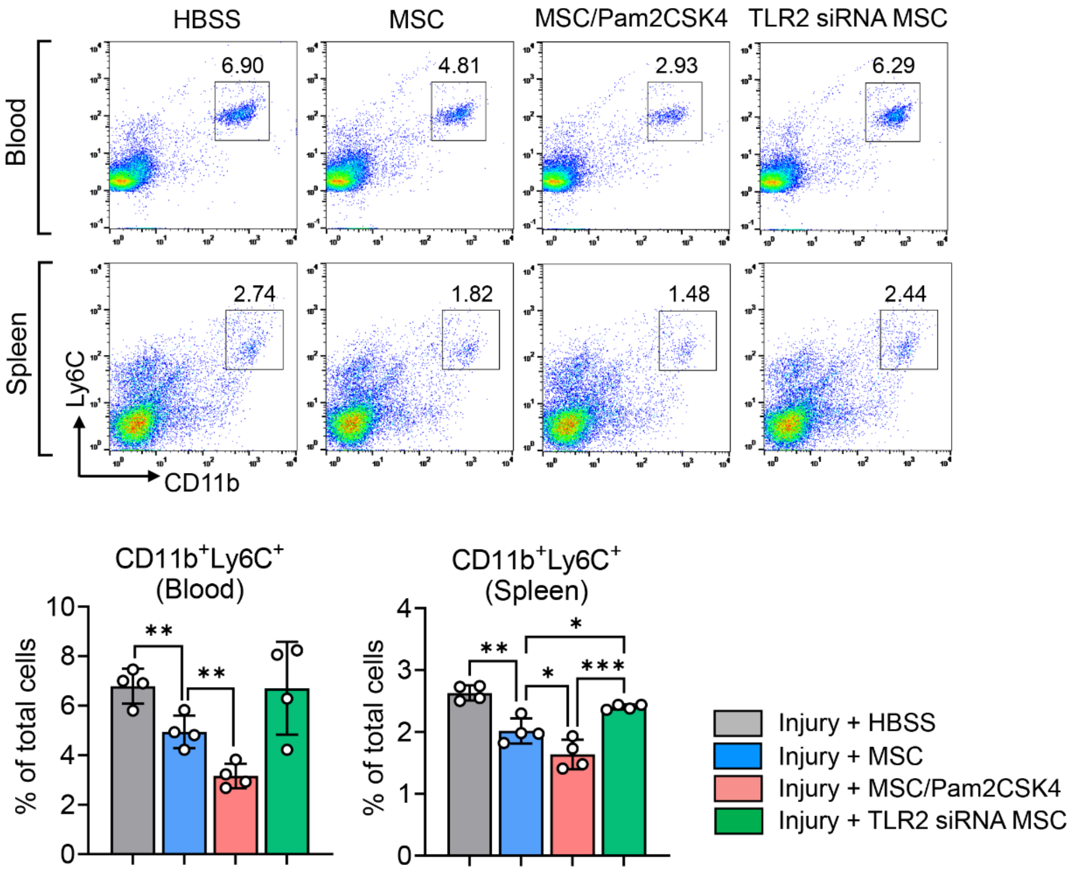

Representative and quantitative flow cytometric analysis for CD11b^{hi}Ly6C^{hi} cells in blood and spleen in BALB/c mice 7 d after corneal suturing injury and treatment with MSCs, Pam2CSK4-pretreated MSCs, TLR2 siRNA-transfected MSCs or HBSS (vehicle). *$p < 0.05$, **$p < 0.01$, ***$p < 0.001$, as analyzed by one-way ANOVA and Tukey's test

Figure S2. Validation of AKR1C1 inhibition in MSCs

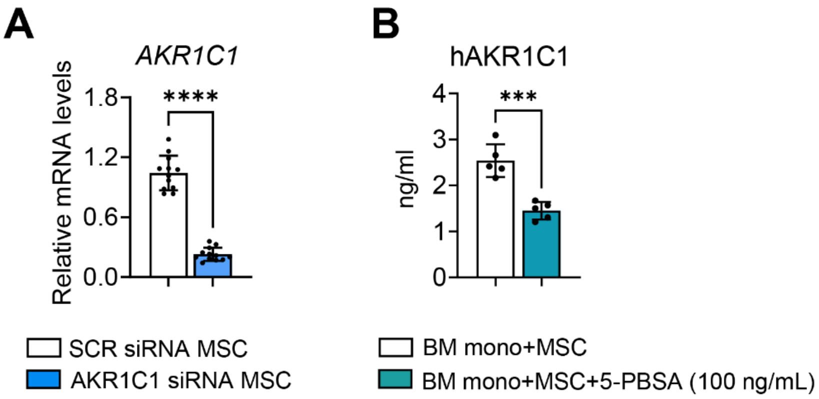

A. qRT-PCR for *AKR1C1* in MSCs transfected with either AKR1C1 siRNA or control SCR siRNA

B. ELISA for AKR1C1 in cell-free supernatants of MSC cultures treated with 5-PBSA, an AKR1C1 inhibitor (100 ng/mL)

Data represent means ± SD. ***$p < 0.001$, ****$p < 0.0001$, as analyzed by Student's *t*-test

Figure S3. AKR1C1 in MSCs partly mediates the cells' action on monocytes and macrophages.

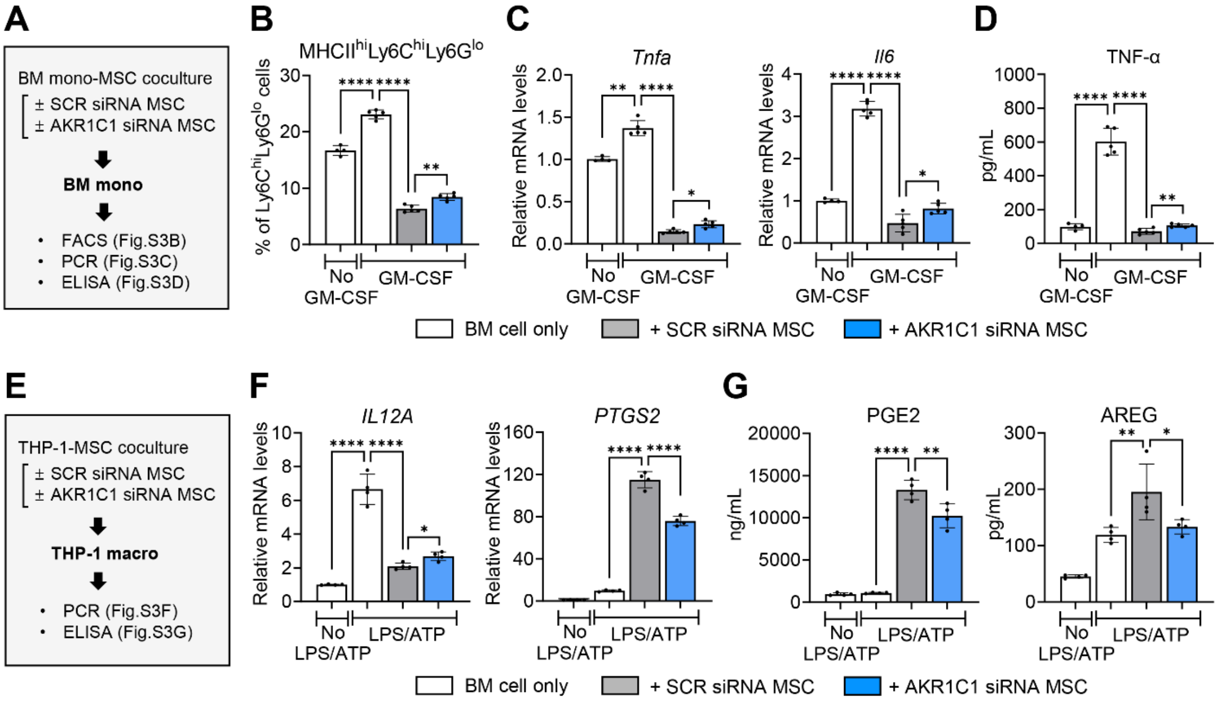

A. Experimental scheme. Under GM-CSF stimulation, murine BM cells were cocultured with MSCs transfected with either AKR1C1 siRNA or SCR siRNA for 5 d. Then BM cells were subjected to assays.

B. Flow cytometry for MHC class II^{hi}Ly6C^{hi}Ly6G^{lo} cells in BM cells

C. qRT-PCR for *Tnfa* and *Il6* in BM cells. The mRNA levels are presented relative to BM cells without GM-CSF treatment.

D. ELISA for mouse-specific TNF-α levels in supernatants of cocultures

E. Experimental scheme. THP-1 macrophages stimulated with LPS/ATP were cocultured with AKR1C1 siRNA-transfected or SCR siRNA-transfected MSCs, and 18 h later, THP-1 macrophages were subjected to assays.

F. qRT-PCR for *IL12A* and *PTGS2* in THP-1 macrophages. The mRNA levels are presented relative to LPS/ATP-unstimulated THP-1 macrophages.

G. ELISA for PGE2 and AREG levels in culture supernatants

Data represent means ± SD. *$p < 0.05$, **$p < 0.01$, ****$p < 0.0001$, as analyzed by one-way ANOVA and Tukey's test, Kruskal-Wallis test and Dunn's multiple-comparison test or Student's *t*-test (SCR siRNA-transfected MSC-treated cells vs. AKR1C1 siRNA-transfected MSC-treated cells in D, *IL12A* in F)
